# Beneficial Effects of Red Wine Polyphenols on Human Health: Comprehensive Review

**DOI:** 10.3390/cimb45020052

**Published:** 2023-01-17

**Authors:** Ivana Buljeta, Anita Pichler, Josip Šimunović, Mirela Kopjar

**Affiliations:** 1 Faculty of Food Technology, Josip Juraj Strossmayer University, F. Kuhača 18, 31000 Osijek, Croatia; 2 Department of Food, Bioprocessing and Nutrition Sciences, North Carolina State University, Raleigh, NC 27695, USA

**Keywords:** polyphenols, red wine, human health, cardiovascular diseases, chemoprevention effect, gut microbiota

## Abstract

Polyphenols are secondary plant metabolites synthesized during the development of the grape berry as a response to stress conditions. They are important constituents in red wines that contribute to the sensory properties and antioxidant activity of wines. Due to the development of highly sophisticated analytical devices, it is now possible to characterize the structure of highly polymerized polyphenols and obtain a full polyphenol profile of red wines. Red wine polyphenols include the ones present in grapes as well as new polyphenol products formed during the winemaking process. Among them, the most important groups and their representatives are flavanols (catechin), stilbenes (*trans*-resveratrol), flavonols (quercetin) and hydroxybenzoic acids (gallic acid). It is known that polyphenols exhibit beneficial effects on human health, such as anti-inflammatory, anticarcinogenic and cardio-protective effects. Many studies have been conducted on the health effects of red wine polyphenols in cancer chemopreventive activities, neuroprotective effects and impact on cardiovascular diseases, gut microbiota in humans, etc. This review will provide major scientific findings on the impact of red wine polyphenols on human health as well as a review of polyphenols present in red wines and their main features.

## 1. Introduction

Wine production dates to a period between 5400 and 5000 Before Common Era and it is still spread all over the world [1]. Wine is a widely consumed alcoholic beverage with pleasant sensory properties, and it is usually fermented from the European species *Vitis vinifera* and American grape species *Vitis labrusca*. During red wine production, the grape juice (must) is fermented at the same time with grape skin and other grape parts. The other processes include fining, filtering and bottling [1,2].

Over 500 compounds have been characterized in wine so far and the main components are sugars, organic acids, alcohols, minerals, pigments, polyphenols, nitrogenous substances, gums, pectins, mucilages, volatile compounds, salts, vitamins and sulfur dioxide [1,3]. Major bioactive compounds in wine are ethanol and polyphenols [3]. Polyphenols are secondary metabolites of plants widely present in fruits, vegetables and their derived products. Among scientists, they attract a lot of attention and were declared as nutraceuticals of modern life [4]. Berries, red wine, whole grains, citrus and green tea are examples of foods rich in polyphenols, and a diet rich with those ingredients is highly recommended due to polyphenols’ positive impact on health [5,6]. The presence of polyphenols affects the taste, palatability and nutritional value of foods. They also have an impact on the characteristics and quality of red wines [7]. In addition to the effect on color and flavor of wine, polyphenols act as natural wine preservatives. A glass of red wine contains around 200 mg of total polyphenols, while in the same quantity of white wine, there is around 30 mg of total polyphenols [3]. Polyphenol composition in one-year-old red wine includes around 5–8% of catechins, 5–10% dimer procyanidins, 10–15% of anthocyanidins, 3–6% phenolic acids, <1% of flavonols, <0.3% of resveratrol and 60–80% polymeric polyphenols [8]. The qualitative and quantitative polyphenol content in red wine depends on different factors, such as environmental factors in the vineyards (soil, climate, exposure to fungal infections), grape varieties and maturity, pre-fermentative practices (addition of sulfur dioxide and ascorbic acid before crushing), fermenting and aging conditions and other technological practices (ionic exchange, filtration, centrifugation, cold stabilization) [9]. It was observed that the delaying of the harvesting date resulted in higher levels of anthocyanins and extractable tannins in wine [1]. The decomposition of grape solids following crushing grapes facilitates the release of polyphenols [10]. At the beginning of winemaking, when a crushing process is carried out, the enzymatic and non-enzymatic reactions start and continue through fermentation and aging [8]. During wine aging, polyphenols from wine transform into complex molecules derived from the condensation of catechins, proanthocyanidins and anthocyanins. As a result, new pigments can form and modifications in wine color occur. New polyphenols, such as vanillin and hydrolysable tannins, during the process of oak aging can appear in the wine. Tannins protect wine from oxidation and they come into oaks from the lignin structure in the wood. Finally, it can be said that wine’s chemical composition is much more complex than the raw material [9].

Previously published scientific studies discussed the role of diet in the prevention of chronic diseases by highlighting dietary intervention on health status and restoring the balance due to previous imbalances caused by lifestyle and environmental factors [11]. Polyphenols possess many biological properties, such as anti-inflammatory responses, antiviral, carcinostatic properties, prevention of low-density lipoprotein oxidation, antihypertensive effect, antimicrobial, antiaging properties, etc. [10,12]. Further, higher polyphenol exposure resulted in lower levels of inflammatory biomarkers [13]. An explanation of some mechanisms for health benefits of polyphenols is their ability to interact with biological systems through modulation of gene expression. Such mechanisms are usually investigated in vitro and in animal models. That includes activation of endothelial nitric oxide production, reduction in tau aggregation and neuroinflammation, interaction with intracellular signaling pathways, etc. [14]. Polyphenols showed a positive impact on lipid metabolism by alleviating lipid metabolism disorders and lowering blood glucose levels [6]. The health-promoting effect of red wine is supported by epidemiological evidence and includes improvements in endothelial dysfunction and hypertension, dyslipidemia and metabolic disorders [11]. Moderate and regular wine consumption is one of the possible reasons for the low rates of coronary heart disease in the French population, despite the high intake of dietary cholesterol and saturated fat. The ”French paradox” increased interest among scientists in the relationship between wine consumption and cardiovascular health [15,16]. Studies have shown correlation between wine consumption and prevention of metabolic syndrome and its related medical complications [17].

Considering the widespread consumption of wine and the significance of its positive effects on human health, this review summarizes the health benefits of polyphenols as well as the main bioactive components in red wine.

## 2. Polyphenols in Red Wine

Polyphenols are classified according to the number of phenol rings and the chemical groups attached to the rings. They are generally made of two aromatic rings connected via a three-carbon bridge and each ring contains at least one hydroxyl group. Simple polyphenols are made of a single aromatic ring with one or more hydroxyl groups attached [9]. Polyphenols are usually divided into flavonoids and non-flavonoids. Flavonols, flavones, flavan-3-ols, flavanones, anthocyanidins, isoflavonoids and dihydrochalcones belong to the group of flavonoids. Non-flavonoids include phenolic acids, tannins and lignans [18].

Polyphenols found in grapes include anthocyanins, flavanols, flavonols, phenolic acids and stilbenes (such as resveratrol and picetannol). They are usually found in the solid parts of grapes (skins, seeds and stems). In addition to proanthocyanidins, which occur as polymers and oligomers, other grape polyphenols are found in monomeric or low-weight forms. In the grape skin, anthocyanins are usually found while flavonoids are mainly found in seeds and stems. The proanthocyanidins are mainly found in seeds, followed by stem and skin, while the pulp is free of them [19].

### 2.1. Phenolic Acids

Phenolic acids are divided into two subgroups: hydroxybenzoic acids and hydroxycinnamic acids. Both subgroups are present in wine. The hydroxybenzoic acid group includes gallic acid, ellagic acid, protocatechuic acid, vanillic acid, *p*-hydroxybenzoic acid and syringic acid derived from benzoic acid [1,20]. Gallic acid is present in red wine but not in grapes. It is assumed that gallic acid is formed by the hydrolysis of tannins [21]. From the group of hydroxycinnamic acids, chlorogenic acid is the main representative, while some others are sinapic acid, coutaric acid, caffeic acid, ferulic acid, caftaric and fertaric acid [1,20].

### 2.2. Resveratrol

Resveratrol (3,5,4′-trihydroxystilbene) is a non-flavonoid polyphenol present in a small number of foods, such as grapes and red wine [22]. Its biosynthesis occurs through the phenylalanine route [1]. It is produced by grapes as a response to stress stimuli, such as injury, ultraviolet exposure or fungal infection [3]. This molecule exists in two isoforms, *cis*- and *trans*-resveratrol. Before the fermentation of grape juice, the major compound is the *cis*-isomer while the final product contains a higher amount of *trans*-isomer [3]. However, other research reported that high concentrations of *cis*-resveratrol were determined in wine while it was not detected in grape juice or skins. The authors suggested its formation by *trans*-resveratrol isomerization or the breakdown of resveratrol polymers during fermentation occurred [23]. The concentration of resveratrol depends on the variety, geographical location maturity, time of harvest and health of the grape [22].

### 2.3. Anthocyanins

Anthocyanins are responsible for the color of some fruits and vegetables as well as their products such as red wine [4]. The chemical structure of anthocyanins is made of three cyclic carbon rings C6–C3–C6, known as a flavylium cation with a positive charge [24]. They are flavonoids present in the form of glycoside and acylglycoside of anthocyanidins. Representatives of anthocyanidins (aglycone) with different hydroxyl or methoxyl groups are cyanidin, delphinidin, malvidin and peonidin. Sugars usually abundant in anthocyanins are glucose, xylose, rhamnose, galactose, fructose and arabinose. Glycosylation and acylation enhance the solubility of anthocyanins and it is known that glycosides are more stable than the corresponding aglycone. Anthocyanins present in red wine are malvidin-3-*O*-glucoside, cyanidin-3-*O*-glucoside, peonidin-3-*O*-glucoside and delphinidin-3-*O*-glucoside [25]. Depending on the pH, anthocyanins vary in color, changing between red and blue due to different chemical forms in an aqueous solution [4,24]. At pH values from 1 to 3, the flavylium cation (red) predominates. By increasing pH values, deprotonation from C7, C4′ and C5 occurs and forms three quinonoidal basic forms (purple). An increase in pH value above 7 causes the formation of secondary deprotonation yielding quinonoidal anions (blue) [24]. In addition to being responsible for the initial color of red wine, anthocyanins are associated with changes that occur during wine aging due to their condensation with flavanols and/or other smaller compounds (vinylphenol, pyruvic acid, glyoxylic acid) [4].

### 2.4. Flavan-3-ols

Flavan-3-ols are one of the most abundant polyphenols in nature, whose structures differ in the stereochemistry of the carbon 3 of ring C and in the hydroxylation degree of ring B. They consist of a monomeric unit ((+)-catechin or (−)-epicatechin) and they can combine to form polymers and proanthocyanidins (condensed tannins). In grape skin tannins, polymers with up to 80 units were found. Further, flavan-3-ols can be esterified with gallic acid (epigallocatechin, epigallocatechin gallate) [4].

### 2.5. Flavonols

Flavonols present in red wine include aglycons, such as quercetin, myricetin, kaempferol and rutin, as well as their glycosides (glucosides, galactosides, glucuronides and diglycosides) [1,21]. Quercetin is one of the most abundant flavonoids present in red wines. Among the flavonoid groups, these compounds are recognized as the main active compounds due to their wide range of biological activities [21].

### 2.6. Tannins

Tannins contribute to the astringency of wine and have the ability to interact and precipitate proteins. They are divided into hydrolyzable and condensed tannins. Hydrolyzable tannins are esters of monosaccharides with gallic acid or oligomers of gallic/ellagic acids [4]. Due to interaction with proteins, tannins can be responsible for the transportation of polyphenols through the body and the expression of their antioxidant potential [4]. Hydrolyzable tannins originate from barrels of wood and are extracted into the wine during wine aging [21].

## 3. Red Wine Polyphenols and Health

**Figure 1:**
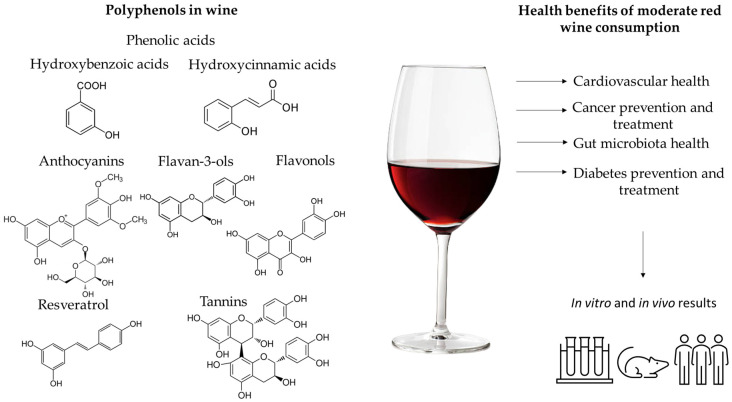
Polyphenols present in red wine and the health benefits of moderate red wine consumption.

In the next several sections, the health benefits of moderate red wine consumption and its polyphenols will be described. Figure 1 presents the main polyphenols present in red wine and their health benefits proven by in vivo and in vitro studies.

### 3.1. Red Wine Polyphenols for Cancer Prevention and Treatment

Cancer is one of the main reasons for death worldwide. It affects more than 6 million people a year [26]. A promising strategy named chemoprevention is defined as the use of natural or synthetic substances or their combinations for blocking, reversing or retarding the process of carcinogenesis [27]. Polyphenols are considered constituents in foods and beverages responsible for reducing the risk of cancer. They are proven to be protective for cell cultures and in animals pre-treated with carcinogenic chemicals or cancer cells [26]. Colorectal cancer affects 1.8 million people each year and it is the third-most-common type of cancer. Most of the diagnoses start as non-cancerous polyps in the intestinal epithelium on the inner lining of the colon or rectum, which accumulated oncogenic mutations over time. These non-cancerous polyps can transform into malignant adenomatous polyps if not detected in time. Among other things, environmental factors, such as diet, smoking, alcohol consumption and a sedentary lifestyle, have a significant impact on this progression. Several studies highlight the benefits of a proper diet (such as the Mediterranean diet) as a protective factor for many diseases, including cancer [28,29]. The consumption of polyunsaturated fatty acids through the Mediterranean diet or food rich in polyphenols has a beneficial effect on cancer prevention [30].

The apoptotic effects of red wine polyphenols on human colon cancer cells (SNU-C4) have been investigated [31]. The results showed that polyphenols (100 µg/mL) increased the apoptosis of SNU-C4 cells through morphological changes in chromatin condensation and apoptotic body formation. In comparison with a control group, it was observed that polyphenols reduced the expression of gene *Bcl-2* and increased the expression of *Bax* and *Caspase-3* genes. It can be suggested that polyphenols have potential as an anti-colon-cancer agent [31]. Polyphenol-rich plant extracts (red wine, pomegranate, white grape and rosemary extracts), as inhibitors of colon carcinogenesis in rats, were investigated [32]. The extracts were added to workshop-made cured meat, whose intake promotes colon carcinogenesis. The supplementation lasted for 14 days for rats and 100 days for azoxymethane-induced rats. The results showed a positive impact of dried red wine, pomegranate extract and α-tocopherol on the decreased number of mucin-depleted foci per colon. It was suggested that the incorporation of these extracts in cured meat can reduce the risk of colorectal cancer that is connected with the consumption of processed meat [32]. Furthermore, the efficiency of red wine extracts on the proliferation of colon cancer cells in vitro and colonic aberrant crypt foci in vivo was investigated [33]. A long vinification process resulted in red wine extracts with superior anti-proliferative activity in tested cells and the prevention ability of the appearance of aberrant crypt foci in mice. Further, it was noticed that quercetin and *trans*-resveratrol showed synergistic anti-proliferative effects [33].

Prostate cancer ranks fourth place worldwide, affecting 1.28 million people each year [28,29]. Factors that cause this disease include radical and ethnic background, age and hereditary genes, as well as environmental and lifestyle factors [28]. The moderate consumption of red wine had a protective effect against prostate cancer while white wine moderate consumption increases the risk of prostate cancer [34]. This may be due to the polyphenols that are mainly found in red wine and their anticancerogenic effects [34].

Anticarcinogenic properties of polyphenols from red wine (quercetin, gallic acid, *trans*-resveratrol and (+)-catechin) were studied [35] and a mouse skin cancer model was employed. Animals were treated with polyphenols (from 0 to 125 mM) twice a week for a total of eighteen weeks topically. The probit analysis results showed that quercetin was the most effective (ED_50_ < 1 µmol), followed by (+)-catechin (ED_50_ 5 µmol), *trans*-resveratrol (ED_50_ 6 µmol) and gallic acid (ED_50_ 5–10 µmol). Further, *trans*-resveratrol was adsorbed more efficiently than quercetin and (+)-catechin in humans after oral consumption. Authors concluded that *trans*-resveratrol might be the most effective anticancer polyphenol in red wines because of highly efficient adsorption after oral consumption in humans [35]. Furthermore, dehydrated–dealcoholized red wine was tested for chemopreventive activities in mice. It was reported that red wine solids supplement delayed tumor onset and that catechin was absorbed by mice. A supplemented diet (red wine polyphenols) supported normal growth and reproduction for three generations, which made it suitable for animal studies and human clinical trials [36]. One study dealt with a comparison of the effects of black and green tea and red wine on azoxymethane-induced intestinal carcinogenesis. Male rats, treated with azoxymethane, were divided into three groups, and to their diet, black tea, green tea or red wine extracts were added. Black tea and wine extract showed a significant impact on fewer colorectal tumors than in controls. These results indicated that black tea and wine extracts could protect against azoxymethane-induced colon carcinogenesis, with a mechanism that involves increased apoptosis in tumors [37]. Selected studies of red wine polyphenols’ impact on cancer are presented in Table 1.

### 3.2. Red Wine Polyphenols and Cardiovascular Health

Coronary heart disease and stroke are the main causes of mortality and disability in developed countries [43]. Most coronary heart diseases are due to atherosclerosis. Based on scientific studies, light-moderate alcohol consumption is associated with a lower occurrence of type 2 diabetes, a higher level of high-density lipoprotein cholesterol and a reduction in lipid oxidative stress. Compared with other alcoholic beverages, red wine is more effective for the prevention of coronary heart disease. It could be that the presence of alcohol together with antioxidants had apparent beneficial properties [21].

Preclinical studies showed that polyphenols have the ability to reduce low-density lipoprotein oxidation [43]. Several mechanisms are involved in red wine’s impact on cholesterol where polyphenols participate in hepatic cholesterol and lipoprotein metabolism. It takes place by reducing cholesterol absorption and decreasing its delivery to the liver and consequently reducing plasma cholesterol. Furthermore, polyphenols have an impact on apolipoproteins A and B, the main factors for cardiovascular disease, as well as on increasing lipoprotein lipase activity and decreasing low-density lipoprotein circulation [44]. One study [45] investigated the effect of moderate consumption of red wine, dealcoholized red wine and gin on glucose metabolism and lipid profile. The study included sixty-seven men with high cardiovascular risk. All participants, for four weeks, received 30 g alcohol/day and, for dealcoholized red wine, the amount was equivalent to red wine. The results showed constant fasting glucose values during the study, a decrease in HOMA-IR (Homeostatic Model Assessment for Insulin Resistance) and mean-adjusted plasma insulin after wine and dealcoholized wine, increased high-density lipoprotein cholesterol, apolipoprotein A-I and A-II after gin and red wine and decreased lipoprotein after red wine intake. Paraoxonase 1 is a hydrolytic enzyme and has a role in high-density lipoprotein protective properties. The impact of red wine consumption on paraoxonase 1 activity in a healthy population was investigated and the results suggested that moderate consumption of red wine positively affected paraoxonase 1 activity in a healthy Mexican population [46]. Subclinical coronary atherosclerosis was investigated by Salazar et al. [13]. The study included mainly male participants and they were subjected to plaque measurements in the carotid and femoral after polyphenol intake. The higher intake of flavonoids was connected with a lower risk of femoral and carotid subclinical atherosclerosis and a higher intake of stilbenes resulted in a lower risk of femoral subclinical atherosclerosis and positive coronary calcium. Chronic intake of red wine polyphenols in 12-, 20- and 40-week-old rats and the possibility of preventing aging-related impairments in vascular function and physical exercise capacity were examined [47]. Rats received red wine polyphenols or apocynin (antioxidant and NDPH oxidase inhibitor) from week 16 to 40. The results showed that both supplementations improved endothelial dysfunction, normalized oxidative stress and the expression of different proteins. As a conclusion, red wine polyphenols defend against aging-induced endothelial dysfunction [47]. Acute consumption of red wine and dealcoholized red wine’s impact on postprandial lipid and lipoprotein metabolism in seventeen dyslipidaemic postmenopausal women was investigated [48]. In this case, acute consumption had no impact on postprandial triglyceride, chylomicrons or insulin homeostasis; in fact, it exacerbated postprandial lipaemia and increased insulin secretion over a six-hour period. Thus, it can be assumed that chronic consumption may be beneficial for cardiovascular disease [48]. In one investigation, moderate red wine drinkers (elderly population) at high cardiovascular risk had a lower risk of developing metabolic syndrome and having abnormal waist circumference, low high-density lipoprotein cholesterol concentrations, high blood pressure and hyperglycemia, compared with non-drinkers [49].

The results of the investigation [50] showed that the estrogen receptor α contributes to the vascular protection of polyphenols and also investigated the contribution of that receptor on the effects of red wine polyphenols on cardiovascular and metabolic alterations associated with obesity. For the investigation, ovariectomized wild-type or estrogen-receptor-α-deficient mice were used. They received standard or Western diets with or without polyphenols for 12 weeks. The results showed that in Western-diet-fed mice, red wine polyphenols reduced plasma triglycerides, adiposity and oxidative stress in the heart, aorta, adipose and liver tissues. Estrogen-receptor-α deletion reduced some beneficial effects of polyphenols [50]. Malvidin-3-*O* glucoside, anthocyanin present in grape skin and red wine, has a role in tumor cell inhibition [51]. Further, one study provided evidence that this compound reduces mammalian myocardial contractility and relaxation and induced coronary vasodilation [51]. Resveratrol has an important role in cardiovascular diseases, by improving endothelial function and glucose metabolism, reducing inflammation and regulating blood lipids [22]. Its benefits on ischemia-reperfusion injuries are through helping in the protection during myocardial infraction. Furthermore, it helps to elicit the expression of many antioxidant enzymes (glutathione peroxidase, superoxide dismutase, catalase and heme oxygenase). Further, it may inhibit the proliferation of liver, oral, breast and prostate cell lines [1]. Selected studies of red wine polyphenols’ impact on cardiovascular diseases are presented in Table 2.

### 3.3. Red Wine Polyphenols and Diabetes

Interest among scientists in compounds with potential anti-diabetic activities is on the rise. Such compounds could be a basis for new drugs used for the treatment and prevention of various diseases [57]. Diabetes mellitus is a complex metabolic syndrome that, according to World Health Organization statistics, will affect about 500 million people by 2025. Complications caused by this disorder include dysfunction of the retina, kidneys, limbs, heart, nerves and blood vessels and, in addition to the violation of life quality, it causes death [57]. Several studies showed that moderate consumption of wine is associated with lower risk of type 2 diabetes [58].

Enriched wine concentrate, with natural polyphenols, modulated the level of hyperglycemia, the normalized concentration of hemoglobin and the number of erythrocytes in experiments with conditions of type 1 diabetes. Treatment with wine concentrate resulted in inhibitions in lipid peroxidation and oxidative modification in proteins in the plasma of rats with experimental diabetes mellitus as well as increased activity of superoxide dismutase and reduced activity of catalase and glutathione peroxidase [57]. The anti-diabetic properties of Portugal red wine were studied in vitro [59]. Four fractions of red wine obtained by solid-phase extraction and dealcoholized red wine were used and results showed all samples had strong inhibitory activities toward α amylase and α glucosidase. The main compounds that are responsible for these activities are monomeric and oligomeric flavan-3-ol compounds [59]. One study dealt with digestion of red wine together with glucose and whey protein food models and results showed that co-digestion affected both wine polyphenol and constituent digestion, bioaccessibility and colonic metabolism. The most important result is that glucose bioaccessibility was reduced, which confirms the hypoglycemic effects related to moderate wine consumption. Further, protein degradation was retarded and short-chain fatty acid production increased (specifically butyric acid) [60]. Selected studies of red wine polyphenols’ impact on diabetes are presented in Table 3.

### 3.4. Red Wine Polyphenols and Gut Microbiota Health Status

It has been found that dietary polyphenols increase the number of beneficial bacteria as well as antimicrobial actions against pathogenic bacteria [67]. The moderate consumption of red wine can have a direct impact on the microbiota and polyphenols can increase the presence of health-related species (*Akkermansia muciniphila* and *Faecalibacterium prausnitzii*) [68]. Nineteen volunteer humans were subjected to an evaluation of red wine polyphenol impact on gut microbiota. Results showed a positive correlation between the total fecal concentration of red wine polyphenols and appearance of *Phascolarctobacterium* sp., *Flavobacterium* sp., *Pelotomaculum* sp., *Prevotella copri* and *Prolixibacter* sp. short-chain fatty acids, which appeared to increase and were in correlation with wine intake [68]. Queipo-Ortuño et al. [69] investigated the effect of red wine polyphenols on selected gut microbial groups. Ten healthy male volunteers received 272 mL per day (20 days) dealcoholized red wine. The results showed a significantly increased number of *Prevotella*, *Enterococcus*, *Bifidobacterium*, *Bacteroides*, *Bacteroides uniformis*, *Eggerthella lenta* and *Blautia coccoides*–*Eubacterium rectale* groups. Further, triglyceride, total cholesterol, high-density lipoprotein cholesterol, C-reactive protein concentrations, systolic and diastolic blood pressures decreased. Moreno-Indias et al. [70] investigated the effect of red wine polyphenols on the modulation of gut microbiota composition and the decreasing risk factors for metabolic syndrome in obese patients. In the investigation, ten healthy subjects and ten metabolic syndrome patients were included. The subjects consumed red wine and dealcoholized red wine for 30 days each (separated by washout period of 15 days). The results showed that the type of wine (normal and dealcoholized) did not affect dominant bacterial composition. The number of fecal bifidobacterial, *Lactobacillus* and butyrate-producing bacteria (*Faecalibacterium prausnitzi* and *Roseburia*) increased in the metabolic syndrome patients. By modulation of gut microbiota with red wine polyphenols, managing metabolic diseases associated with obesity could be achieved.

Gastrointestinal infections are a serious public-health problem due to mortality worldwide and increasing resistance to antibiotics [71]. New strategies, such as a diet rich in potential therapeutic compounds, may help with the treatment of these infections [71]. Adhesion of bacteria to intestinal epithelial cells and production of toxins cause their pathogenicity in the intestine [72]. As polyphenols can protect plants from pathogenic microorganisms, they are investigated to be used as antimicrobial agents for therapeutic purposes. *Escherichia coli* is normal intestinal microbiota, but some strains can cause serious diseases in the intestinal tract or in the urinary tract. The role of red wine polyphenol extract in the protection of the human colonic epithelial cell line against one strain of *Escherichia coli* (*Escherichia coli* 270) was tested. The results showed that *Escherichia coli* 270 adheres to intestinal epithelial cells and secretes an exotoxin and that red wine polyphenol protected cells from death. The mechanisms did not include inhibition of the adhesion of *Escherichia coli* to the cells but inhibition of action of a protein toxin seems to have occurred [71].

### 3.5. The Role of Red Wine Polyphenols in Oral Health

Oral health is an important element in general health and affects life quality. Dental caries, edentulism, periodontal disease and oral cancer are oral diseases and poor mouth hygiene is the main risk factor for oral diseases [73]. The oral microbiota are represented by more than 700 species [74]. Interest in natural-origin therapies for the maintenance of oral health is on the rise. Polyphenols may be used as new strategies for the prevention and treatment of oral pathologies through antimicrobial, antiadhesive or anti-inflammatory activity [74].

Several studies showed that wine polyphenols can modulate the composition of the oral microbiota and have prevented caries and periodontal diseases [75]. The results of one study [76] showed inhibition of adhesion and biofilm formation of *Streptococcus mutans* with a high-molecular-weight polyphenol fraction of red wine (proanthocyanidins). Selected wine polyphenols (*p*-coumaric and caffeic acids), grape seed and red wine extracts were used for investigation of their effects on *Fusobacterium nucleatum*, *Porphyromonas gingivalis* and *Streptococcus mutans* adherence to human gingival fibroblasts. The results showed partial inhibition of *Streptococcus mutans* adhesion to human gingival fibroblast after treatment with p-coumaric and caffeic acids, while for *Fusobacterium nucleatum*, caffeic acid, p-coumaric acid and red wine polyphenol extracts showed inhibitory action. *Porphyromonas gingivalis* adhesion was inhibited with all used extracts and individual compounds [74]. The antimicrobial properties of red wine in an oral biofilm model have been investigated [77]. Five species, usually associated with oral disease, were used for the biofilm model of the supragingival plaque, such as *Actinomyces oris*, *Streptococcus mutans*, *Fusobacterium nucleatum*, *Veillonella dispar* and *Streptococcus oralis*. The results showed that red wine and dealcoholized red wine application reduced the *Fusobacterium nucleatum* and *Streptococcus oralis* population [77].

### 3.6. Red Wine Polyphenol Neuroprotective Properties

Several studies investigated resveratrol’s neuroprotective properties. In neuronal stem cells, pretreated with resveratrol, activated nuclear factor erythroid 2-related factor 2 and protection of cells from oxygen–glucose deprivation were observed [78]. A resveratrol metabolite, piceatoannol, showed protection of HT22 neuronal cells from glutamate-induced cell death [79]. In rats, supplemented with resveratrol and then induced cerebral ischemic injury, a decrease in oxidation biomarkers and reestablished superoxide dismutase activity due to resveratrol treatment were observed [80]. Glutathione is the most important antioxidant in brain cells and enzymes; glutathione peroxidase and glutathione reductase are essential to maintain its functionality. Studies showed that red wine consumption increased the activity of these enzymes [14]. A study by Rocha-Parra et al. [81] showed a protective effect of red wine powder (freeze-dried with maltodextrin and gum arabic) on human neuroblastoma SH-SY5Y cell viability co-incubated with 6-hydroxydopamine. Indeed, 150 µg/mL of red wine powder, in a concentration of 1479 ng GAE/mL, exhibited the most protective effect against 6-hydroxydopamine cytotoxicity and ensured 88.3% surviving cells. Polyphenols that have multiple hydroxyl groups have the ability to capture α-dicarbonyl species and scavenge reactive oxygen species that caused the formation of mono- and di-adducts inhibiting advanced glycation end products. In that way, neurodegenerative diseases can be prevented [82].

### 3.7. Red Wine Polyphenols and Red Blood Cells

Production of oxygen-derived free radicals is connected to the onset of many diseases (cancer, atherosclerosis, rheumatoid arthritis). Reactive oxygen species are involved in the damage process of red blood cells and sickle cell anemia. Many defense mechanisms are developed in living organisms to limit reactive oxygen species levels and their effect [10]. The plasma membrane redox system represents the first protection mechanism from oxidative stress to neutralize plasma free radicals in human erythrocytes. It was proved that several polyphenols (resveratrol, quercetin and myricetin) can potentiate cellular antioxidant systems (including plasma membrane redox system). Human erythrocytes, treated with red wine polyphenols (73 µg/mL gallic acid equivalents), resulted in increased glutathione intracellular concentration [10]. That increase depends upon the activation of glutathione reductase and glucose-6-phosphate dehydrogenase whose enzymatic activities increase by about 30% and 47%, respectively. Changes in the glutathione pathway caused by red wine polyphenols were connected to an increase in reactive oxygen species. Therefore, the authors concluded that the pro-oxidant effect of red wine polyphenols caused an adaptive stress response in erythrocytes, enhancing their antioxidant defense [10]. A similar study on the plasma membrane redox system (PMRS) showed that the anthocyanin fraction of red wine had the capacity to positively modulate PMRS enzymatic activity [83]. The effect of two red wine types (Aglianico and Novello) and one white wine on red blood cells was examined [10]. Under experimentally set conditions, H_2_O_2_ caused red blood cell lysis. It was observed that Aglianico (the sort with a higher concentration of polyphenols) provided 25% inhibition in red blood cell hemolysis. Further, preincubation of red blood cells with that wine made a protective effect against oxidative stress (intracellular malondialdehyde maintained at a level comparable to cells without the oxidative stress) [10]. Via isolation of a specific class of polyphenols from red wine, anthocyanins, their protective effect on red blood cells against reactive oxygen species damage was evaluated. Firstly, the authors demonstrated that the tested red wine and its fractions show no toxic effect on red blood cells. Red blood cells are prone to oxidative damage due to a high level of hemoglobin, which is a promoter of oxidative processes. The results showed that fractions containing anthocyanins had strong antioxidant properties (lower reactive oxygen species levels) and lower methemoglobin production in human red blood cells with micromolar doses of H_2_O_2_ [25]. A group of post-myocardial infarct patients was included in a two-week study, receiving red wine (250 mL per day) or water. The results showed that moderate red wine consumption, even for a short period, has a positive effect on blood parameters, such as increased erythrocyte membrane fluidity and antioxidant status [84]. A group of non-smoking male volunteers (39 participants) was supplemented with 200 mL per day of red wine or water for 3 weeks. In the group that received red wine, whole blood viscosity significantly decreased while the hematocrit/whole blood viscosity ratio increased. Further, red blood cell aggregation decreased and its deformability at high shear stress increased [85].

## 4. Conclusions and Future Perspectives

In this review, the impact of red wine polyphenols on human health was discussed and, more specifically, its impact on cardiovascular disease, cancer prevention and treatment, gut microbiota, oral health and diabetes. From the reviewed animal and human studies, it can be concluded that there is a high potential in the polyphenols of wine, i.e., in its moderate consumption. Among scientists, there is no consensus on whether these health benefits are due to ethanol or polyphenols’ presence in red wine. However, it can be concluded from all the above-mentioned studies that polyphenols are responsible to a greater extent for these properties. Future studies should be more focused on investigating the impact of red wine polyphenols on the prevention and treatment of some other diseases.

## Figures and Tables

**Table 1:** Selected studies of red wine polyphenols’ impact on cancer.

Source of Polyphenols	Experimental Model	Outcomes	Conclusions	Reference
Red wine polyphenols	F344 rats were fed for 16 weeks with 50 mg/kg of red wine polyphenols. Colon carcinogenesis was induced with a total dose of 7.4 mg/kg of azoxymethane or 300 mg/kg of dimethylhydrazine.	The used dose of polyphenols inhibited colon carcinogenesis and these animals had lower cancer yield than the control rats. The main microorganisms in the feces of polyphenols-treated rats were *Lactobacillus*, *Bacteroides*, and *Bifidobacterium* spp. While in control-fed rats feces were *Bacteroides*, *Propionibacterium*, and *Clostridium* spp.	Red wine polyphenols can cause a reduction of oxidative damage, variation in gene expression and modulation of colonic flora.	[38]
Red wine concentrate	Red wineconcentrate, its total polyphenolic extract, purified epicatechin, catechin, resveratrol, and quercetin were tested on the proliferation of hormone-sensitive (MCF7, T47D) andresistant (MDA-MB-231) breast cancer cell lines.	The results showed that the picomolar or nanomolar range of polyphenols can decrease proliferation in a time- and dose-dependent manner.	Consumption of wine due to the presence of polyphenols (even in low concentrations in the human body) could have a beneficial antiproliferative effect on breast cancer cell growth.	[39]
Resveratrol, ferulic acid, epicatechin, ellagic acid, rutin, chlorogenic acid, coumalic acid, vanillic acid, syringic acid, morin, phloridzin	The lung adenocarcinoma cell lines HOP62 and H1975 were used for the examination of the anti-lung cancer activity of red wine polyphenols. HOP62 cells were injected in female BALB/C nude mice and then treated with ellagic acid.	The results showed that ellagic acid inhibited lung cancer cell proliferation with efficiency similar to resveratrol. Tumor-bearing mice, treated with ellagic acid resulted in significantly inhibited tumor growth with suppressed CIP2A levels and increased autophagy.	Ellagic acid has the potential as a lung cancer chemotherapy agent, especially in combination with celastrol.	[40]
Red wine extract	For the study, human colorectal cancer cell lines SW620, HCT116, murine colorectal cancers CT26 and MC38, and rat non-transformed small intestinal IEC-18 cells were used.	The results showed that red wine extract reduces colorectal cancer cells in vitro. In vivo study showed that red wine extract caused a decrease in colorectal tumor growth associated with a decrease in tumor-infiltrating lymphocytes.	From the obtained results, it can be concluded that mixtures of polyphenols can play a role in modulating the immune response and, consequently, inflammation.	[41]
Cabernet Sauvignon and Rombola wine polyphenols extracts	Prostate cancer cells were treated with red or wine extracts in concentration range from 15 to 1000 µg/mL.	The results showed that red wine and white wine extracts have impact on proliferation, survival, oxidative status and induction of autophagy of prostate cancer cells.	The results give insight into the implications when designing a more effective adjunct treatment for prostate cancer patients.	[42]

**Table 2:** Selected studies of red wine polyphenols’ impact on cardiovascular diseases.

Source of Polyphenols	Experimental Model	Outcomes	Conclusions	Reference
Sicilian red wine	48 subjects consumed 250 mL of wine per day for 4 weeks.	The results showed that LDL/HDL, factor VII, fibrinogen, plasma C-reactive protein, oxidized LDL antibody significantly decreased. HDL-C, plasminogen activator inhibitor antigen, apolypoproteins A1, transforming growth factor-β1, tissue plasminogen activator antigen and total plasma antioxidant capacity increased.	Moderate consumption of red wine in the adult population is suggested due to its positive effect on many risk factors and inflammatory biomarkers.	[52]
Grape- wine extract and grape juice extract	60 subjects with high systolic blood pressure were treated with placebo capsules, capsules with a mixture of grape and wine extract and capsules with grape juice extract alone for 10 weeks.	The results showed that grape-wine extract consumption decreased 24-h ambulatory systolic/diastolic blood pressures and a decrease in plasma concentrations of the vasoconstrictor endothelin-1 by 10% was observed. No effects on blood pressure and other parameters for grape juice extract alone were observed.	It can be assumed that presence of catechins and procyanidins in grape-wine extract may contribute to this blood pressure lowering effect.	[53]
Red wine and gin	40 healthy men with mean age 38 years; 28 days received 30 g ethanol/day and 15-day washout period.	Compared to gin intervention, red wine intake reduced plasma superoxide dismutase activity and malondialdehyde levels; lag phase time of low-density lipoprotein oxidation analysis increased 11 min after wine, compared to gin whereas no differences were observed.	Red wine intake has greater antioxidant effects compared to gin probably due presence of polyphenols.	[54]
Red wine	60 male Wistar rats (45 days old) were used for the study. They were divided into two groups and fed with a standard diet or westernized diet. After changes in mass and glycemic levels animals received red wine, water or hydroalcoholic solution.	From the results it was observed that obese animals with presented alteration in the cholesterol, triglycerides and serum levels of glucose that received red wine had improvement in these metabolic profiles, while that was no case with animals that received hydroalcoholic solution.	It can be concluded that moderate and chronic use of red wine improves the glycemic, lipid and oxidative stress profile in rats fed with an obesogenic diet.	[55]
Red wine and ethanol	Wistar Kyoto rats, diabetic streptozotocin-induced Wistar Kyoto rats and spontaneously hypertensive rats were treated with ethanol (12.5%) 3.715 mL/kg/day, red wine (12.5%) 3.715 mL/kg/day or NaCl 0.9% (as control) for 3 weeks.	After treatment with red wine, a reduction of systolic blood pressure from diabetic and spontaneously hypertensive rats occurred.	The study has shown that red wine may have a beneficial effect on the cardiovascular system.	[56]
Lyophilized red wine *RIO SOL* Cabernet-Sauvignon	6-week treatment of spontaneously hypertensive rats with 100 or 300 mg/kg/day intra-gastrically.	Reduced blood pressure and smooth muscle hypercontractility, decreased eutrophic remodeling and vascular collagen deposition, reduced platelet aggregation.	These findings refer that tested wine had a cardiovascular protective effect in spontaneously hypertensive rats by decreasing oxidative stress.	[15]

**Table 3:** Selected studies of red wine polyphenols’ impact on diabetes.

Source of Polyphenols	Experiment Conditions	Outcomes	Conclusions	Reference
Red wine	The studied group includes 17 type 2 diabetes patients treated with low doses of oral hypoglycaemic agents or with diet only. The first group of 9 patients received 360 mL of red wine per day (divided for lunch and dinner) for two weeks and second group of 8 patients did not consumed wine (control diabetics).	The results showed that red wine consumption improved insulin-mediated whole-body glucose disposal by 43%.	Red wine consumption for 2 weeks significantly weakens insulin resistance in type 2 diabetic patients, without affecting vascular reactivity and nitric oxide production.	[61]
Resveratrol	Male Wistar rats were divided in normal and diabetic groups and sacrificed. The rental artery samples were treated with resveratrol.	The results showed that different subtypes of K channels engage in resveratrol effect on the rental artery of diabetic rats.	Resveratrol manifests a relaxant effect on the renal artery of diabetic and normal rats.	[62]
Red wine	The studied group include 18 diabetic patients and 13 healthy controls that received 300 mL of red wine for three weeks.	The red wine consumption decreased serum hepcidin in both groups without significant changes in serum ion, soluble transferrin receptors and transferrin saturation.	Examining the effect of red wine consumption on hepcidin, which is a key regulator of iron metabolism and acute-phase protein, provides insight into the mechanisms of the cardiometabolic benefits of moderate wine consumption, especially in diabetic patients.	[63]
Polyphenol extract from Corbières red wine	Streptozocin-induced diabetic rats or healthy control rats were used 6 weeks-treatment with red wine polyphenol extract, ethanol or both.	Polyphenols treatment reduced body growth, food intake and glycemia in control and diabetic rats. In diabetic rats supplemented with ethanol or ethanol-polyphenol combination, body growth was partially restored, and hyperglycemia was reduced.	Polyphenol extract reduces glycemia in diabetic and nondiabetic rats and ethanol or ethanol-polyphenols combination can correct the diabetic state.	[64]
Red wine	9 participants (3 women and 6 men) with either type 2 diabetes or pre-diabetes received 263 mL of water or red wine. 30 min after consumption, participants started an oral glucose tolerance test in which blood samples were taken periodically for 3 h.	Consumption of red wine caused an increase in the incremental area under the curve for glucose-dependent insulinotropic peptide by 25% and for insulin by 50%, while for glucose and glucagon-like peptide 1 no differences were observed.	Acute red wine consumption doesn’t seem to be effective for enhancing glycemic control or maybe need to be combined with therapy for improvement of insulin sensitivity.	[65]
Red wine polyphenols	Twenty Wistar rats weighing 200 to 220 g were subjected to a high-fat diet for 2 months. Then they were divided into 2 groups: those that received only a high-fat diet and those who received a high-fat diet and red wine polyphenols (50 mg/kg) for an additional 2 months. 10 control rats were subjected to a normal diet for 4 months.	Rats subjected to a high-fat diet increased body weight (over 20%) as well as blood levels of glucose, C-peptide, oxidized proteins and lipid peroxides. Red wine polyphenols weakened oxidative stress due to high-fat diet in plasma, tissue and islet cell hyperplasia without impact on blood glucose levels and hepatic steatosis.	These results showed a positive impact of red wine polyphenols against metabolic syndrome and supported the use of polyphenols in treatments for diabetic patients.	[66]

## Data Availability

Data presented in manuscript.
